# Bi-lineage B- and T-lymphoid Extramedullary Blast Crisis at an Initial Presentation of Chronic Myeloid Leukemia: A Case Report and Literature Review of Extramedullary Blast Crisis

**DOI:** 10.7759/cureus.49348

**Published:** 2023-11-24

**Authors:** Naoufal Benlachgar, Azlarab Masrar, Soukaina Haidouri, Hicham Harmouche, Zoubida Tazi Mezalek

**Affiliations:** 1 Department of Clinical Hematology, Ibn Sina Hospital, University Mohamed V of Medicine, Rabat, MAR; 2 Central Laboratory of Hematology, Ibn Sina Hospital, University Mohamed V of Medicine, Rabat, MAR; 3 Department of Internal Medicine, Ibn Sina Hospital, University Mohamed V of Medicine, Rabat, MAR

**Keywords:** tyrosine kinase inhibitors (tkis) therapy, t-cell acute lymphoblastic leukemia, b-cell acute lymphoblastic leukemia, chronic myeloid leukemia (cml), extra medullary blast crisis

## Abstract

Chronic myeloid leukemia (CML) with BCR-ABL1-positive cells is a myeloproliferative neoplasm (MPN) characterized by a chromosomal translocation t(9,22)(q34.1;q11.2), which results in the formation of a Philadelphia (Ph) chromosome containing the BCR-ABL1 fusion gene. Extramedullary blast crisis (EBC) associated with bcr/abl-positive CML is a rare initial presentation.

Here, we present and discuss the case of a 51-year-old man who presented with a weight loss history, cervical swelling, and left-sided abdominal pain. He had a white blood cell count of 147,910/mm3. The blood smear study revealed myelemia in 23% and 8% of blast-like cells. The bone marrow aspiration and biopsy showed a richly cellularized sample; the megakaryocytes were present; the granular neutrophil line was at 89% with blasts at 1%. The cytogenetic analysis revealed a complex karyotype with the presence of a Philadelphia chromosome t (9, 22) (q34, q11) associated with additional cytogenetic abnormalities (ACA). Molecular analysis (PCR) detected a BCR::ABL1 (p210) rearrangement. At this point, a diagnosis of CML in the chronic phase was confirmed, but a cervical lymph node biopsy analysis revealed a bi-phenotypic B/T-lymphoblastic lymphoma (LBL) and expressed at fluorescent in situ hybridization (FISH) analysis BCR::ABL1 rearrangement. These findings were consistent with the diagnosis of a bi-phenotypic B/T extramedullary blast crisis associated with CML.

## Introduction

Chronic myeloid leukemia (CML) with BCR-ABL1-positive is a myeloproliferative neoplasm (MPN) characterized by a chromosomal translocation t(9,22)(q34.1;q11.2), which results in the formation of a Philadelphia (Ph) chromosome containing the BCR::ABL1 fusion gene [1]. This gene encodes for a tyrosine phosphokinase protein that stimulates predominantly granulocyte production and leads to genomic instability. Extramedullary blast crisis (EBC) associated with BCR::ABL1-positive CML is a rare initial presentation [1]. The prognosis for EBC is poor, and there is no recommendation for the treatment of this subtype of CML. Here, we present a case of a patient with a simultaneous diagnosis of CML associated with a bi-lineage (B and T) lymphoblastic lymphoma (LBL) at initial presentation and discuss the EBC associated with CML.

## Case presentation

A 51-year-old man presented with a weight loss history, cervical swelling, and left-sided abdominal pain in February 2021. These symptoms started five months prior to the first consultation. On examination, the patient was found to have splenomegaly as well as cervical, axillary, and inguinal lymphadenopathy. He had a white blood cell count of 147,910/mm^3^ and hemoglobin at 10.7 g/dl without thrombocytopenia. The blood smear study revealed myelemia at 23%, metamyelocytes at 8%, 4% of promyelocytes, a lymphocyte count of 10,350/mm3, eosinophils at 1480/mm^3^, basophils at 7400/mm^3^, and 8% of blast-like cells.

**Figure 1:**
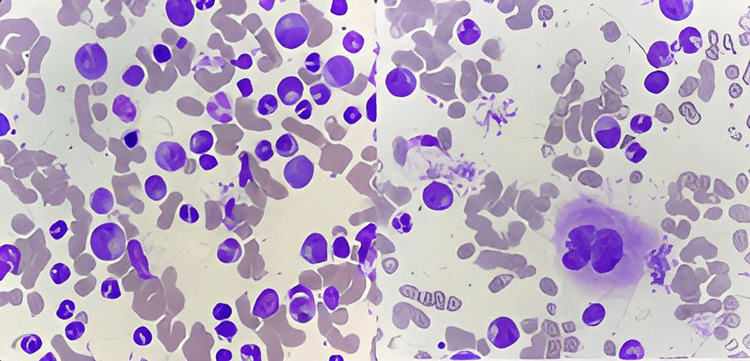
Bone marrow aspiration analysis Bone marrow aspiration shows hypercellular bone marrow with myeloid predominance; blasts are not increased. The findings are consistent with chronic myeloid leukemia, chronic phase (MGG stain, original magnification 1000). Iconography Central Hematology Laboratory, Ibn Sina Hospital Rabat: Pr. Azlarab Masrar.

The bone marrow aspiration and biopsy showed a richly cellularized sample; the megakaryocytes were present; the granular neutrophil line was at 89% with blasts at 1%, promyelocytes at 9%, myelocytes at 8%, metamyelocytes at 12%, polynuclear neutrophils at 59%, and basophils at 3% (Figure 1).

The flow cytometry analysis detected the presence of 4.4% of a myeloid blast in the bone marrow. The cytogenetic analysis revealed a complex karyotype with the presence of a Philadelphia chromosome t (9, 22) (q34, q11) in 6 out of the 30 mitoses analyzed associated with a trisomy: 6, 8, 10, 15, 19, 21. Molecular analysis (PCR) detected a BCR-ABL1 (p210) rearrangement. Table 1 reports the most relevant characteristics at diagnosis.

**Table 1:** The most relevant characteristics at diagnosis

Data	March 2021	Reference range
Hb (g/dl)	10.7	13–17
Leukocyte count/mm^3^	147,910	4500–11,000
Platelet count/mm^3^	247,000	150,000–400,000
Peripheral blast %	8	-
Bone marrow blast %	1	-
Karyotype	t (9,22) in q34, q11 + ACA	46, XY
Blast % by flow cytometry on BM	4.4	-
Molecular analysis (RT-PCR)	BCR-ABL1 rearrangement	-

At this point, a diagnosis of CML in the chronic phase was made with SOKAL RR 1.36 (high) and ELTS 2.1576 (intermediate-risk group). The patient started a first-generation tyrosine kinase inhibitor (TKI), Imatinib mesylate, at a dose of 400 mg per day. The presence of a generalized lymphadenopathy was of great concern given the risk of an associated lymphoma or an active infectious disease.

**Figure 2:**
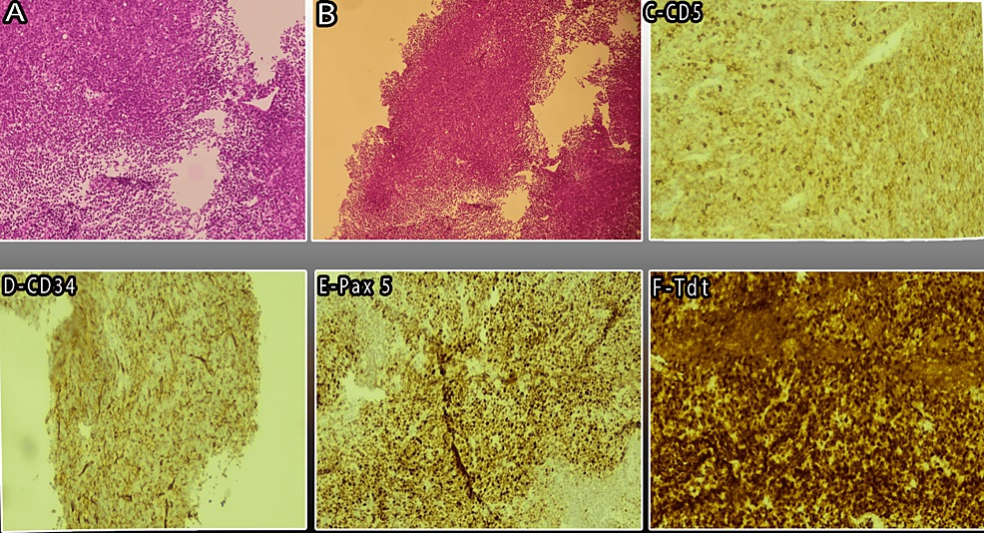
Histopathology of the lymph node biopsy (A-B) H&E staining shows tumoral proliferation consisting of medium-sized cells with high nucleocytoplasmic ratios and nuclei with fine chromatin reminiscent of blast cytology. Mitoses were noted as well as small foci of necrosis. (C-F) The immunohistochemistry study showed that the bi-phenotypic features of this blastic infiltrate express both a B-phenotype characterized by expression of PAX5 (E) (CD20-negative) and a T-phenotype characterized by expression of CD5 (C). There were expressions of the immature lymphoid markers Tdt (F) and CD34 (D). Iconography, Anatomic Pathology Laboratory, Ibn Sina Hospital Rabat.

A cervical lymph node biopsy showed tumoral proliferation consisting of medium-sized cells with high nucleocytoplasmic ratios and nuclei with fine chromatin reminiscent of blast cytology. Mitoses were noted as well as small foci of necrosis. The immunohistochemistry study showed a bi-phenotypic character of this blastic infiltrate, which expresses both a B-phenotype characterized by the expression of CD79a, CD19, CD38, and PAX5 (CD20 were negative) and a T-phenotype characterized by the expression of CD2, CD3, CD5 (subject to background noise) and CD7. There were expressions of Tdt, CD34, and CD33 (subject to background noise). MPO, CD4, and CD8 were negative, as well as CD117 and CD1a. In addition, CD30, MUM1, cyclin D1, and CD10 were negative. The fluorescent in situ hybridization (FISH) analysis on the lymph node biopsy revealed BCR::ABL1 (p210) rearrangement on the blast cells. These findings were consistent with diagnosing a bi-phenotypic B/T-lymphoblastic lymphoma (LBL) (Figure 2; Table 2).

**Table 2:** Immunohistochemistry markers of the lymph node biopsy

Markers	Results
Myeloid markers	
CD34	+
CD33	+ (subject to background noise)
MPO	−
B-cell markers	
CD10	−
CD79a	+
CD19	+
PAX5	+
CD20	−
Cyclin D1	−
MUM1	−
T-cell markers	
CD2	+
CD3	+
CD5	+ (subject to background noise)
CD7	+
Non-specific markers	
CD30	−
CD38	+

The PET/CT had shown stage 3 Ann Arbor lymphoma with supradiaphragmatic and subdiaphragmatic lymph node involvement but no hypermetabolic activity. The LDH value was within the normal range. At one month, the patient showed a complete hematologic response and a partial reduction of the lymphadenopathy (Table 3).

**Table 3:** Comparison of peripheral blood cell count results at diagnosis and at one month’s post-imatinib therapy showing complete hematologic response

Data	March 2021	After 1 month of imatinib (400 mg per day)
Hemoglobin (g/dl)	10.7	13.2
Leukocyte count/mm^3^	147,910	3410
Platelet count/mm^3^	247,000	177,000
Blast percentage on blood smear %	8	0

Given the partial clinical reduction of the lymphadenopathy and the absence of hypermetabolic activity on the PET scan, the decision was to continue imatinib. However, at three months, the patient presented a clinical progression of lymphadenopathy. At this point, we wanted to start a high-dose chemotherapy (HD CMT) regimen with a second-generation TKI, but unfortunately, the patient died before that. The cause of death had not been confirmed, but the initial admission to the ICU was an acute respiratory distress syndrome associated with a SARS-CoV-2 infection.

## Discussion

Extramedullary blast proliferation is defined as an extramedullary proliferation of blast that originates from the CML Ph+; BCR::ABL1+ clone [1]. EBC is frequently diagnosed in the late-stage evolution of CML, not responding to TKI. Nevertheless, some rare cases of EBC associated with CML in the chronic phase at diagnosis have been reported. The most common type of blast crisis phenotype is myeloid and B-lymphoid leukemia/lymphoma [2]. T-lymphoid or mixed phenotypes are extremely rare, and few cases have been reported in the literature [2,3]. Our case is a rare and first-described presentation of a bi-lineage B- and T-extramedullary blast crisis at the initial diagnosis of CML.

Cytogenetic abnormalities associated with blast transformation include trisomy 8 and 19, isochromosome 17, t (3;21), and del17p [4]. The most frequent ACA observed in BC is ACA +8, +Ph, I (17q), +19, +21, +17 [5]. In our case, the karyotype showed three abnormalities of the most frequent ACA in BC (+8, +19, +21). Somatic mutations associated with blast evolution include RUNX1 (33.3%), ASXL1 (20.5%), and IKZF1 (17.9%) [6]. Branford et al. reported six mutations (ASXL1, BCORL1, RUNX1, GATA2, MLL, and UBE2A) that were recurrently mutated in patients with CML and early blast transformation [4].

EBC can involve the skin, lymph nodes, liver, central nervous system, etc. [7,8]. In our case, the blast crisis affected only the lymph nodes. The most important step in the diagnosis of EBC is to regroup the arguments that stand for a common clone of origin for the bone marrow and extramedullary proliferation (Table 4).

**Table 4:** Arguments for EBC/CML and De novo bcr/abl positive acute leukemia/lymphoma [14,15]

Characteristics	EBC/CML	De novo BCR::ABL1 positive acute leukemia/lymphoma
Bone marrow involvement with blast	-	+
Type of BCR::ABL1 breakpoint mutation	Major BCR breakpoint	Minor BCR breakpoint mutations (e1a2 BCR-ABL)
TCR gene rearrangement mutations	-	+ (T cell phenotype)
Age group	Adults	Children or adolescent
CML history	+	-
BCR::ABL1 positive cell	Leukemia and non-leukemia clone cells	Leukemia clone cells
Response to 2ND G TKI + HD CMT	Excellent	-

Conventional cytogenetic analysis (RT-PCR) or Southern blotting on lymph nodes can lead to false-positive BCR::ABL1 results [9,10]. The best tool to confirm the common clone origin is the identification of BCR::ABL1 rearrangements by fluorescent in situ hybridization (FISH) on the blast cells [10]. In our case, the FISH analysis on the lymph node biopsy confirmed the common origin and the bi-lineage phenotype was confirmed by the presence of T markers on blasts such as CD2, CD3, CD5, CD7, and B markers such as CD79a, CD19, CD38, and PAX5.

Otherwise, lymphoblastic lymphoma is FDG-avid in the majority of cases [11]. Shinto et al. reported, in a cohort of 23 LBL, a positive PET at diagnosis in 22 patients. The PET negativity in our case could be explained by a negative result secondary to imatinib therapy started 1 month before. Early response of tumor uptake on 18 F-FDG-PET had been reported in a case of non-hematological malignancy, gastrointestinal stromal tumor (GIST) [12].

Differential diagnoses of lymphadenopathy associated with CML include infection (tuberculosis...), myeloid proliferation (myeloid sarcoma/chloroma), Hodgkin lymphoma (HL), non-Hodgkin lymphoma (NHL) B/T, and lymphoblastic lymphoma B/T-LBL [13]. The prognosis of blast crisis and EBC is very poor [14].

Even with HD chemotherapy, TKI, and SCT, the relapse is frequent [15]. Only 12% of reported cases are alive at the moment of publication [16]. Treatment options for EBC at the initial presentation of CML are described in the literature, and their outcomes are summarized in Table 5.

**Table 5:** Treatment options of EBC (T-cell lymphoblastic lymphoma/leukemia) at the initial presentation of CML described in the literature and their outcomes BM: bone marrow, CML: chronic myeloid leukemia, EBC: extramedullary blast crisis, HD CMT: high-dose chemotherapy, HU: hydroxy-carbamide, m: months, MMR: major molecular response, SCT: stem cell transplantation; TKI: tyrosine kinase inhibitor, PR: partial remission. *Imatinib was started nine months after HD CMT. **Imatinib was started at relapse post-SCT. ^+^Dasatinib started at relapse prior to SCT. ^++^Duration of the follow-up from diagnosis to the last medical record up date.

Author	N	Age (years)/sex	CML phase	EBC phenotype	EBC site	TKI	HD CMT	SCT	Other therapy	Outcomes	Duration of the follow-up (months)^++^
Raanani et al. [3]	1	45/M	BP	T-ALL	BM	Imatinib**	HD CMT	Yes	DLI-Rx	Dead	60
Jin et al. [10]	1	40/M	CP	T-LBL	Nodal	-	-	No	HU	Dead	28
Fu et al. [13]	1	43/M	CP	T-LBL	Nodal	Imatinib	Hyper-CVAD/MOAP	Yes	-	Alive	98
Fu et al. [13]	1	44/M	CP	MS/T-LBL	Nodal	Imatinib*	Hyper-CVAD	Yes	-	Dead	15
Jain et al. [14]	3	63 (2M/1F)	-	3 T-LBL	Nodal	Dasatinib 1 none TKI 2	Hyper-CVAD	1 pt	-	2 alive 1 dead	25
Padhi et al. [15]	1	49/M	CP	T-LBL	Nodal	Dasatinib	Hyper-CVAD	Yes	-	Alive	5
Zeng et al. [22]	1	44/M	CP	T-LBL	Nodal	Imatinib	3 CHOP	Yes	-	Alive	51
Qing et al. [32]	1	32/F	CP	Mixed phenotype (T/B/Myeloid)	Nodal	Dasatinib	Hyper-CVAD	-	-	-	-
Xu and Li [33]	2	24/F; 66/M	BP; CP	T-ALL; T-ALL	BM nodal	Imatinib dasatinib	Hyper-CVAD	Yes 2^nd^ line	HU HD CMT	Dead	30

In the pre-TKI era, the most commonly used treatment to treat the CML blast crisis was HD chemotherapy. T-cell blast transformation seems more sensitive to chemotherapy when compared to myeloid and B-cell phenotypes [17,18]. In the era pre-TKI, HD CMT resulted in a 68% response rate [19].

In patients with CML BP, imatinib mesylate alone resulted in 55% ORR, 25% CR, and 16% CYRR, but the responses were transient and short in most patients [20,21]. CML patients treated with imatinib can develop EBC [2]. Based on this observation, imatinib was expected to have low efficacy in EBC [22].

First-generation TKI imatinib is the most commonly used TKI, but second-generation TKI dasatinib can bring an advantage, especially if CNS involvement is present, and nilotinib can have more potent TK inhibition activity than imatinib [23]. As reported in Table 5, most cases reported in the literature received HD chemotherapy with a first- or second-generation TKI followed by an allo-SCT.

Ponatinib (a third-generation potent oral tyrosine kinase inhibitor of un-mutated and mutated BCR::ABL1) was evaluated in a phase 2 study that included 62 patients with blast-phase CML; 31% had a major hematologic response and 23% had a major cytogenetic response [24]. A higher response rate is achieved when ponatinib is associated to HD chemotherapy (FLAG-IDA regimen) [25].

Ponatinib and Blinatumomab (bispecific T-cell engager anti-CD19 and anti-CD3) associations had been tested in a phase 2 study for the treatment of Philadelphia Chromosome-positive ALL, including five patients with CML in lymphoid blast phase (LBP). The CR/CRi rate was 100% in CML-LBP patients, and 40% achieved complete molecular response [26].

In a phase 1/2 study combining inotuzumab ozogamicin (CD22-directed humanized monoclonal antibody conjugated to the potent cytotoxin) with bosutinib for 18 patients (R/R Ph+ ALL, n=16; LBP-CML, n=2), complete response with incomplete count recovery was achieved in 15/18 (83%) patients; 11/18 (61%) patients achieved negative measurable residual disease by flow cytometry. After a median follow-up of 44 months, the median duration of response and overall survival were 7.7 months and 13.5 months, respectively [27].

Another emerging strategy for relapsed/refractory myeloid blast transformation is the association of Venetoclax (a BCL2 inhibitor) and TKI. In a retrospective study, nine patients with CML in the myeloid blast phase (MBP) received Venetoclax combined with TKI-based regimens. The overall response rate (ORR) and overall survival (OS) were 75% and 10.9 months, respectively [28].

In a retrospective case series of patients with bi-lineage myeloid and lymphoid neoplasms, hematopoietic stem cell transplantation (HSCT) resulted in a statistically significant advantage in overall survival [13]. Allo-SCT is associated with a high rate of transplantation-related mortality [16]. Jiang et al. reported in a retrospective cohort of patients with CML-BC a transplant-related mortality rate of 31.6±7.7% [29]. Despite these adverse events, allo-SCT remains the only available therapy for long-term remission or cure [23].

Hehlmann proposed to treat primary blast crisis CML with imatinib and to change to a second-generation TKI according to the kinase domain mutation profile. In the case of second-generation TKI resistance, ponatinib and an assessment for allo-HCT will be indicated. Ponatinib failure is an indication of an early allo-HCT [30].

For future directions, in patient-derived CML cell lines and murine Ba/F3 cells harboring BCR::ABL1 T315I or T315I-including compound mutations, the association of asciminib (an ABL1 inhibitor) + ponatinib + hydroxyurea produces synergistic apoptosis-inducing effects in CD34+/CD38-CML stem cells obtained from patients with chronic phase CML or BCR::ABL1 T315I+ CML blast phase [31].

## Conclusions

EBC is a rare initial presentation of CML. The prognosis is poor. A few cases of the T-LBL phenotype and one case of the mixed myeloid/T/B phenotype have been reported in the literature. Our case is unique because of the first described phenotype, T/B-LBL, associated with CML at the initial presentation. The limits of this case report are the lack of somatic high-risk mutations in next-generation sequencing.

## References

[1] Inverardi D, Lazzarino M, Morra E (1990). Extramedullary disease in Ph’-positive chronic myelogenous leukemia: frequency, clinical features and prognostic significance. Haematologica.

[2] Kim AS, Goldstein SC, Luger S, Van Deerlin VM, Bagg A (2008). Sudden extramedullary T-lymphoblastic blast crisis in chronic myelogenous leukemia: a nonrandom event associated with imatinib?. Am J Clin Pathol.

[3] Raanani P, Trakhtenbrot L, Rechavi G (2005). Philadelphia-chromosome-positive T-lymphoblastic leukemia: acute leukemia or chronic myelogenous leukemia blastic crisis. Acta Haematol.

[4] Branford S, Wang PP, Parker WT (2015). High incidence of mutated cancer-associated genes at diagnosis in CML patients with early transformation to blast crisis. Blood.

[5] Johansson B, Fioretos T, Mitelman F (2002). Cytogenetic and molecular genetic evolution of chronic myeloid leukemia. Acta Haematol.

[6] Grossmann V, Kohlmann A, Zenger M (2011). A deep-sequencing study of chronic myeloid leukemia patients in blast crisis (BC-CML) detects mutations in 76.9% of cases. Leukemia.

[7] Hyun BH, Gulati GL, Ashton JK (1990). Myeloproliferative disorders. Classification and diagnostic features with special emphasis on chronic myelogenous leukemia and agnogenic myeloid metaplasia. Clin Lab Med.

[8] Sakakura M, Ohishi K, Nomura K (2004). Case of chronic-phase chronic myelogenous leukemia with an abdominal hematopoietic tumor of leukemic clone origin. Am J Hematol.

[9] Ichinohasama R, Miura I, Takahashi N (2000). Ph-negative non-Hodgkin's lymphoma occurring in chronic phase of Ph-positive chronic myelogenous leukemia is defined as a genetically different neoplasm from extramedullary localized blast crisis: report of two cases and review of the literature. Leukemia.

[10] Jin GN, Zou P, Chen WX, Ding ZY, Zhou H (2013). Fluorescent in situ hybridization diagnosis of extramedullary nodal blast crisis. Diagn Cytopathol.

[11] Fox TA, Carpenter B, Taj M (2021). Utility of 18F-FDG-PET/CT in lymphoblastic lymphoma. Leuk Lymphoma.

[12] Shinto A, Nair N, Dutt A, Baghel NS (2008). Early response assessment in gastrointestinal stromal tumors with FDG PET scan 24 hours after a single dose of imatinib. Clin Nucl Med.

[13] Fu X, Shang Y, Zhang L (2018). Analyses and treatment of simultaneous bi-lineage malignancies of myeloid leukemia and lymphoma: two case reports and a literature review. Oncol Lett.

[14] Jain P, Kantarjian H, Jabbour E (2017). Clinical characteristics of Philadelphia positive T-cell lymphoid leukemias-(De novo and blast phase CML). Am J Hematol.

[15] Padhi P, Topalovski M, El Behery R, Cantu ES, Medavarapu R (2018). A rare case of chronic myelogenous leukemia presenting as T-cell lymphoblastic crisis. Case Rep Oncol Med.

[16] Apfelbeck U, Hoefler G, Neumeister P, Fonatsch C, Linkesch W, Sill H (2000). Extramedullary T cell lymphoblastic transformation of chronic myeloid leukaemia successfully treated with matched unrelated donor bone marrow transplantation. Bone Marrow Transplant.

[17] Cervantes F, Villamor N, Esteve J, Montoto S, Rives S, Rozman C, Montserrat E (1998). 'Lymphoid' blast crisis of chronic myeloid leukaemia is associated with distinct clinicohaematological features. Br J Haematol.

[18] 18] Nathwani AC, Goldman JM (1993). Management of chronic myeloid leukaemia in lymphoid blast transformation. Haematologica.

[19] Aguayo A, Cortes JE, Kantarjian HM (1999). Complete hematologic and cytogenetic response to 2-amino-9-beta-D-arabinosyl-6-methoxy-9H-guanine in a patient with chronic myelogenous leukemia in T-cell blastic phase: a case report and review of the literature. Cancer.

[20] Druker BJ, Sawyers CL, Kantarjian H (2001). Activity of a specific inhibitor of the BCR-ABL tyrosine kinase in the blast crisis of chronic myeloid leukemia and acute lymphoblastic leukemia with the Philadelphia chromosome. N Engl J Med.

[21] Kantarjian HM, Cortes J, O'Brien S (2002). Imatinib mesylate (STI571) therapy for Philadelphia chromosome-positive chronic myelogenous leukemia in blast phase. Blood.

[22] Zeng DF, Chang C, Li JP, Kong PY, Zhang X, Gao L (2015). Extramedullary T-lymphoblastic blast crisis in chronic myelogenous leukemia: a case report of successful diagnosis and treatment. Exp Ther Med.

[23] Hehlmann R (2012). How I treat CML blast crisis. Blood.

[24] Cortes JE, Kim DW, Pinilla-Ibarz J (2013). A phase 2 trial of ponatinib in Philadelphia chromosome-positive leukemias. N Engl J Med.

[25] Copland M, Slade D, McIlroy G (2021). Ponatinib in combination with FLAG-IDA chemotherapy for blast-phase chronic myeloid leukemia: final results of the seamless phase I/II dose-finding UK trials Acceleration Programme (TAP) matchpoint trial. Blood.

[26] Short NJ, Kantarjian H, Konopleva M (2021). Updated results of a Phase II study of ponatinib and blinatumomab for patients with Philadelphia chromosome-positive acute lymphoblastic leukemia. Blood.

[27] Jain N, Maiti A, Ravandi F (2021). Inotuzumab ozogamicin with bosutinib for relapsed or refractory Philadelphia chromosome positive acute lymphoblastic leukemia or lymphoid blast phase of chronic myeloid leukemia. Am J Hematol.

[28] Maiti A, Franquiz MJ, Ravandi F (2020). Venetoclax and Bcr-Abl tyrosine kinase inhibitor combinations: outcome in patients with Philadelphia chromosome-positive advanced myeloid leukemias. Acta Haematol.

[29] Jiang H, Xu LP, Liu DH (2014). Allogeneic hematopoietic SCT in combination with tyrosine kinase inhibitor treatment compared with TKI treatment alone in CML blast crisis. Bone Marrow Transplant.

[30] Hehlmann R (2020). Chronic myeloid leukemia in 2020. Hemasphere.

[31] Gleixner KV, Filik Y, Berger D (2021). Asciminib and ponatinib exert synergistic anti-neoplastic effects on CML cells expressing BCR-ABL1T315I-compound mutations. Am J Cancer Res.

[32] Qing X, Qing A, Ji P, French SW, Mason H (2018). Mixed phenotype (T/B/myeloid) extramedullary blast crisis as an initial presentation of chronic myelogenous leukemia. Exp Mol Pathol.

[33] Xu J, Li S (2014). Unusual T-lymphoblastic blast phase of chronic myelogenous leukemia. Case Rep Hematol.

